# Interobserver agreement among midwives in cardiotocography interpretation using the 2015 FIGO guidelines for intrapartum fetal monitoring: A retrospective study

**DOI:** 10.1002/ijgo.70687

**Published:** 2025-11-24

**Authors:** Serena Neri, Ruben Ramirez Zegarra, Elisa NicolinI, Francesca Frati, Sara Tagliaferri, Tullio Ghi

**Affiliations:** ^1^ Midwifery School, Department of Medicine and Surgery University of Parma Parma Italy; ^2^ Unit of Obstetrics and Gynecology, Department of Medicine and Surgery University of Parma Parma Italy; ^3^ Division of Clinical Epidemiology, Department of Clinical Research University Hospital Basel, University of Basel Basel Switzerland; ^4^ Department of Medicine and Surgery University of Parma Parma Italy; ^5^ Catholic University of Sacred Heart Department of Women and Child Health, Fondazione Policlinico Gemelli Rome Italy

**Keywords:** childbirth, intrapartum care, midwifery, observer variation, obstetric labor

## Abstract

**Objective:**

To evaluate the interobserver agreement among midwives for the classification of intrapartum cardiotocography (CTG) tracings and their main parameters—that is, fetal heart rate baseline, variability and decelerations—according to the 2015 FIGO guidelines.

**Methods:**

This was a retrospective, single center observational study conducted at the Maternity Hospital of the University of Parma, Italy between January and September 2024. We selected 100 non‐consecutive intrapartum CTG tracings before operative delivery. A total of six participating midwives (3 senior and 3 junior) received the last 60 min of CTG recordings and classified the trace at the moment of the decision for the operative delivery. The main outcome was the interobserver agreement of the overall interpretation of the CTG tracings. Secondary outcomes included the interobserver agreement for each individual CTG parameter. The agreement between the observers was estimated through the Fleiss' kappa (*κ*).

**Results:**

The overall interobserver agreement for CTG interpretation was fair (κ = 0.35, 95% confidence interval [CI]: 0.31–0.38). The highest agreement was observed for the normal category (*κ* = 0.50, 95% CI: 0.45–0.55), while suspicious and pathologic classifications showed lower agreement (*κ* = 0.25, 95% CI: 0.20–0.30 and 0.37, 95% CI: 0.32–0.42, respectively). Among CTG parameters, decelerations achieved the highest agreement (*κ* = 0.48, 95% CI: 0.43–0.53), followed by baseline rate (*κ* = 0.43, 95% CI: 0.39–0.47). In contrast, agreement on variability was poor (*κ* = 0.20, 95% CI: 0.16–0.24).

**Conclusion:**

The overall interobserver agreement for intrapartum CTG interpretation using the 2015 FIGO guidelines was fair. Agreement for individual parameters varied widely.

## Abstract 2

Our study shows that when interpreting intrapartum CTG tracings using the 2015 FIGO Guidelines midwives' agreement is fair. While there is better agreement for “normal” classifications, consensus is lower for “suspicious” and “pathologic” tracings. Among individual parameters, decelerations and variability had the highest and the lowest agreement respectively.

## 1 INTRODUCTION

Cardiotocography (CTG) was introduced in the 1960s as a tool for intrapartum fetal monitoring to reduce perinatal mortality and the incidence of cerebral palsy. However, despite its widespread use, CTG has not achieved this goal; instead, it has been associated with an increase in cesarean section and operative vaginal delivery rates.^1^, ^2^ One of the main hypotheses is that CTG interpretation is highly subjective resulting in poor to moderate—at best—intra‐ and interobserver agreement.^3^, ^4^, ^5^, ^6^, ^7^

One of the most widely adopted guidelines for intrapartum CTG interpretation is the 2015 FIGO (the International Federation of Gynecology and Obstetrics) guideline.^8^ It classifies CTG tracings into three categories—normal, suspicious or pathologic—based on three main parameters: fetal heart rate baseline, variability, and the presence of repetitive decelerations.^9^ While some evidence suggests that the 2015 FIGO guideline may yield better intra‐ and interobserver agreement than other CTG guidelines,^10^, ^11^, ^12^ its reliability remains suboptimal. Most studies on the intra‐ and interobserver agreement have been focused on obstetricians and medical trainees.^13^

In many countries (particularly in Europe), midwives are usually the primary caregivers for labouring women, making their ability to interpret CTG crucial for clinical decision making. To date, only two studies have assessed the intra‐ and interobserver agreement for CTG interpretation among midwives. Devane et al. evaluated the intra‐ and interobserver agreement using the 1987 FIGO guidelines,^14^ while our research group^15^ compared the interobserver agreement of CTG interpretation according to the 2015 FIGO guidelines with the physiology‐based CTG guidelines.^16^

The aim of the present study was to assess the interobserver agreement among midwives for the classification of intrapartum CTG tracings and its main parameters based on the 2015 FIGO guidelines.

## 2 MATERIALS AND METHODS

This retrospective, single center observational study was conducted at the Maternity Hospital of the University of Parma, Italy between January and November 2024. The study protocol was approved by the local Ethics Committee (699/2022/OSS/UNIPR). This study was reported following the Guidelines for Reporting Reliability and Agreement Studies (GRAAS).^17^

We employed 100 non‐consecutive intrapartum CTG tracings, which had been used in a previous study.^15^ Briefly, these tracings were selected from women who underwent operative delivery (cesarean section or instrumental vaginal delivery) for various obstetrical indications, including suspected fetal hypoxia, labor arrest in the first or second stage, and non‐hypoxic intrapartum injury. Therefore, not all CTG tracings were necessarily considered abnormal by the attending clinicians at the time of delivery. All CTG traces were pseudonymized before analysis: patient identifiers were removed, and each trace was labeled with a unique numeric code. Only the researchers responsible for trace selection had access to identifiable data.

Inclusion criteria were singleton term pregnancies in the active phase of labor (defined as cervical dilation >6 cm), with a fetus in cephalic presentation, with no fetal malformations, and an indication for continuous CTG monitoring. Exclusion criteria were multiple pregnancies and poor CTG signal quality. Continuous fetal monitoring was performed until delivery, with a standard paper speed of 1 cm/min. All CTG tracings were anonymized before the analysis.

Six clinical midwives actively working in the labor ward volunteered to participate in the study. They were stratified based on professional experience into three senior midwives (more than 5 years of professional experience) and three junior midwives (less than 5 years of professional experience). Since 2016, the University Hospital of Parma has adopted the 2015 FIGO guidelines as the standard reference for intrapartum CTG interpretation. Consequently, all midwives working in the labor ward had received practical training and dedicated courses on these guidelines through internal meetings. Before the assessment, all participants were provided with written copies of the FIGO 2015 guidelines and instructed to base their evaluations strictly on these criteria to ensure comparable baseline understanding.

The participants received a digital recording of the last 60 min of the CTG tracing prior to the decision for operative delivery. They visually interpreted and independently classified the CTG tracings at the time of the decision for the operative delivery, following the 2015 FIGO guidelines.^9^ Midwives were not given a time limit for CTG interpretation and were allowed to consult the guidelines during the assessment. The individual fetal heart rate parameters (i.e., baseline, variability, decelerations) were assessed and based on this, the overall CTG trace was classified as normal, suspicious, or pathologic. All the participating midwives were blinded to demographic, clinical, labor and neonatal outcomes.

Participants were initially asked to classify the fetal heart rate baseline and variability into three categories: normal, suspicious or pathologic. Decelerations were initially assessed simply as either present or absent. However, during an internal meeting held a few months after the initial evaluation, we concluded that a more detailed classification would offer greater clinical relevance. Therefore, a second evaluation round was conducted using a more detailed subclassification based on the 2015 FIGO guideline to ensure consistency across participating midwives.

In this second step, cases in which decelerations were present were re‐evaluated using five subcategories: absent, repetitive decelerations lasting <3 min, repetitive decelerations lasting >3 min, late decelerations, and single prolonged deceleration lasting >5 min. Unfortunately, two senior midwives declined to participate in this second assessment. As a result, only three junior midwives and one senior midwife completed the re‐evaluation.

All data were recorded and stored in a secured, pseudonymized Microsoft Excel spreadsheet (Microsoft Corporation, Redmond, WA, USA), with access restricted exclusively to the research team. The main outcome was the interobserver agreement of the overall interpretation of the CTG tracings. Secondary outcomes included the interobserver agreement for each individual CTG parameter and the impact of clinical experience on CTG interpretation.

### 2.1 Statistical analysis

Statistical analyses were performed using the Statistical Package for the Social Sciences (SPSS), version 29 (IBM Corp.) and R version 4.3.2. Sample size estimation, defined as the number of CTG tracings required, was determined through a simulation study. It was estimated that 100 CTG tracings would be needed to achieve a confidence interval width of 0.19, corresponding to the width of an interpretation category for interobserver agreement.

The level of interobserver agreement was assessed using Fleiss' kappa (*κ*), which quantifies agreement among multiple observers across categorical outcomes. Kappa values were calculated for the overall CTG classification, as well as for each individual CTG parameter (baseline, variability, decelerations). For multi‐category variables, *κ* values were calculated both globally and within each category to assess agreement at different clinical subcategories. Confidence intervals (CI) at 95% were reported for all *κ* estimates.

Fleiss' kappa coefficients with 95% confidence intervals were computed for junior and senior midwives subgroups. Differences in agreement were evaluated based on the overlap of confidence intervals; non‐overlapping intervals were interpreted as evidence of a statistically significant difference. Strength of the agreement was interpreted as follows: very good (*κ* ≥ 0.81), good (*κ* = 0.61–0.80), moderate (*κ* = 0.41–0.60), fair (*κ* = 0.21–0.40), and poor (*κ* ≤ 0.20).

## 3 RESULTS

The baseline and perinatal characteristics of the women, from whom the 100 CTG tracings were selected, have been previously reported elsewhere and are summarized in Table S1. The mean maternal age was 32.4 ± 5.4 years, and 75% of the women were nulliparous. Cesarean section was performed in 40% of cases, with suspected intrapartum fetal compromise being the most frequent indication for operative delivery (46%). A cord pH <7.1 was observed in seven cases (7.0%), and an Apgar score <7 at 5 min was recorded in five cases (5.0%).

**FIGURE 1:**
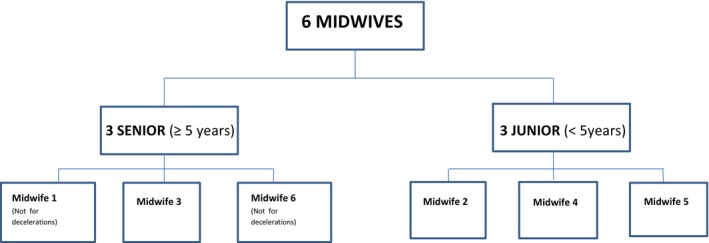
Flow chart of reviewers based on years of experience.

A total of six midwives independently interpreted 100 intrapartum CTG tracings, classifying the overall trace, as well as the fetal heart rate baseline and variability, according to the 2015 FIGO guidelines. Only four midwives (3 junior and 1 senior) assessed decelerations (Figure 1). Table 1 summarizes the interobserver agreement for the overall CTG interpretation and each individual parameter. The overall interobserver agreement for CTG interpretation was fair (*κ* = 0.35 [95% CI: 0.31–0.38]). Agreement was moderate for CTG tracings classified as “normal” (*κ* = 0.50 [95% CI: 0.45–0.55]), and fair for those classified as “suspicious” (*κ* = 0.25 [95% CI: 0.20–0.30]) and “pathologic” (*κ* = 0.37 [95% CI: 0.32–0.42]).

**TABLE 1:** Interobserver agreement for overall and individual CTG parameter categories, according to FIGO guidelines (2015).

Variable	Fleiss' kappa [95% CI]
CTG classification	0.35 [0.31–0.38]
Normal	0.50 [0.45–0.55]
Suspicious	0.25 [0.20–0.30]
Pathologic	0.37 [0.32–0.42]
Fetal heart rate baseline	0.43 [0.39–0.47]
Normal	0.54 [0.49–0.59]
Suspicious	0.42 [0.37–0.47]
Pathologic	0.13 [0.08–0.18]
Fetal heart rate variability	0.20 [0.16–0.24]
Normal	0.22 [0.17–0.27]
Suspicious	0.31 [0.26–0.36]
Pathologic	−0.07 [−0.12 to − 0.01]
Decelerations^a^	0.48 [0.43–0.53]
Absent	0.71 [0.63–0.79]
Repetitive decelerations lasting <3 min	0.43 [0.35–0.51]
Repetitive decelerations lasting >3 min	0.34 [0.26–0.42]
Late decelerations	0.36 [0.28–0.44]
Single prolonged deceleration lasting >5 min	0.57 [0.49–0.65]

Abbreviations: CI, confidence interval; CTG, cardiotocography; FIGO, the International Federation of Gynecology and Obstetrics.

^a^ Based on four midwives' evaluation.

Among the individual CTG parameters, overall assessment of the fetal heart rate baseline showed moderate interobserver agreement (*κ* = 0.43 [95% CI: 0.39–0.47]). Agreement was moderate for both the “normal” (*κ* = 0.54 [95% CI: 0.49–0.59]) and “suspicious” (*κ* = 0.42 [95% CI: 0.37–0.47]) categories, but poor for the “pathologic” category (*κ* = 0.13 [95% CI: 0.08–0.18]). For fetal heart rate variability, the overall interobserver agreement was poor (*κ* = 0.20 [95% CI: 0.16–0.24]). When examining individual categories, agreement was fair for both the “normal” (*κ* = 0.22 [95% CI: 0.17–0.27]) and “suspicious” (*κ* = 0.31 [95% CI: 0.26–0.36]) classifications. However, agreement for the “pathologic” category was poor (*κ* = −0.07 [95% CI: −0.12 to −0.01]).

Decelerations exhibited the highest interobserver agreement among all CTG parameters, with an overall moderate agreement (*κ* = 0.48 [95% CI: 0.43–0.53]). When analyzing individual categories, agreement was good for the “absent” category (*κ* = 0.71 [95% CI: 0.63–0.79]). Moderate agreement was observed for “single prolonged deceleration lasting >5 min” (*κ* = 0.57 [95% CI: 0.49–0.65]) and “repetitive decelerations lasting <3 min” (*κ* = 0.43 [95% CI: 0.35–0.51]). Agreement was fair for both “repetitive decelerations lasting >3 min” (*κ* = 0.34 [95% CI: 0.26–0.42]) and “late decelerations” (*κ* = 0.36 [95% CI: 0.28–0.44]).

To evaluate the potential impact of professional experience on interobserver agreement, we calculated the Fleiss' kappa separately for junior and senior midwives (Table 2). Interobserver agreement for overall CTG classification was classified as moderate among junior (*κ* = 0.55 [95% CI: 0.46–0.64]) and poor among senior midwives (*κ* = 0.18 [95% CI: 0.09–0.26]). Junior midwives demonstrated good agreement for fetal heart rate baseline evaluation (*κ* = 0.61 [95% CI: 0.51–0.71]), whereas senior midwives showed fair agreement (*κ* = 0.32 [95% CI: 0.23–0.41]). For fetal heart rate variability, agreement was fair among junior midwives (*κ* = 0.21 [95% CI: 0.12–0.30]) and poor among seniors (*κ* = 0.14 [95% CI: 0.06–0.23]). Finally, we did not perform a comparison between junior and senior midwives for the classification of decelerations, as only one senior midwife participated in the re‐evaluation of decelerations.

**TABLE 2:** Interobserver agreement for individual CTG parameter categories, according to FIGO guidelines in midwives with more than 5 years of work experience or with less than 5 years of work experience (>5 years = senior midwives; <5 years = junior midwives).

Variable	Fleiss' kappa [95% CI]		
	Senior midwives	Junior midwives	
CTG classification	0.18 [0.09–0.26]	0.55 [0.46–0.64]	^a^
Normal	0.41 [0.29–0.52]	0.65 [0.54–0.77]	^a^
Suspicious	0.07 [−0.04 to 0.18]	0.47 [0.36–0.59]	^a^
Pathologic	0.16 [0.05–0.27]	0.57 [0.46–0.69]	^a^
Fetal heart rate baseline	0.32 [0.23–0.41]	0.61 [0.51–0.71]	^a^
Normal	0.46 [0.35–0.58]	0.68 [0.56–0.79]	n.s.
Suspicious	0.23 [0.12–0.34]	0.64 [0.53–0.75]	^a^
Pathologic	0.16 [0.05–0.27]	0.21 [0.10–0.33]	n.s.
Fetal heart rate variability	0.14 [0.06–0.23]	0.21 [0.12–0.30]	n.s.
Normal	0.23 [0.12–0.34]	0.20 [0.09–0.31]	n.s.
Suspicious	0.14 [0.03–0.26]	0.40 [0.28–0.51]	^a^
Pathologic	−0.07 [−0.19 to −0.04]	−0.12 [−0.23 to −0.01]	n.s.

*Note*: n.s. Not statistically significant.

Abbreviations: 95% CI, 95% confidence interval; CI, confidence interval; CTG, cardiotocography; FIGO, the International Federation of Gynecology and Obstetrics.

^a^ Statistically significant.

## 4 DISCUSSION

The main findings of the present study were as follows: (1) interobserver agreement for overall CTG classification among midwives was fair, with slightly higher (moderate) agreement for the “normal” category; (2) agreement for individual CTG parameters varied widely, ranging from poor (fetal heart rate variability) to moderate (decelerations); and (3) junior midwives demonstrated higher interobserver agreement than senior midwives across the overall classification and most individual parameters.

Over the years, several national and international guidelines have been developed to standardize CTG interpretation. Among them, the 2015 FIGO guidelines have become one of the most widely adopted standard references.^8^ However, most studies—mainly conducted among obstetricians—have reported that these guidelines yield only fair levels of agreement for overall intrapartum CTG classification.^5^, ^11^, ^12^ In a previous study from our research group involving midwives using the 2015 FIGO guidelines, Neri et al. assessed 100 CTG tracings and reported suboptimal agreement, consistent with our findings. However, in that study the Krippendorff's alpha rather than Fleiss' kappa was used, which does not allow us to directly compare results.^15^

Higher interobserver agreement is often observed for more frequently occurring CTG patterns, such as “normal” tracings, which in our study reached moderate agreement. In contrast, the “suspicious” and “pathologic” categories showed lower agreement, with “suspicious” tracings exhibiting the lowest agreement. This is likely due to the vague definition in the 2015 FIGO guidelines, where “suspicious” CTG tracings do not meet the criteria for “normal” but are also not clearly “pathologic”, making interpretation highly subjective and variable.

Our results are supported by the findings of Martí Gamboa et al.^7^ who evaluated interobserver agreement between two experienced observers across 302 CTG tracings. The authors reported moderate agreement for the “normal” category and fair agreement for “suspicious” tracings, with kappa values comparable to ours. However, their study found moderate agreement for “pathologic” CTGs, whereas we demonstrated only fair agreement. A key difference is that Martí Gamboa et al. included only two observers, likely contributing to a more consistent classification.

For fetal heart rate baseline, our results are consistent with the existing literature, demonstrating moderate and fair agreement when assessed using the 2015 FIGO guidelines.^5^, ^18^, ^19^ Contrarily, interobserver agreement for fetal heart rate variability was poor in our study. Previous studies using various CTG classification systems have consistently reported fair agreement for variability.^5^, ^18^, ^19^ Using the 2015 FIGO guidelines, Rei et al. found fair agreement (*κ* = 0.30) among obstetricians—a higher level than observed in our results. However, their study also noted that agreement decreased when assessing reduced variability, which aligns with our findings.

Decelerations showed the highest interobserver agreement in our study, with overall moderate agreement, and good agreement for the “absent” category. The study by Rei et al. reported a lower agreement for the “absent” category—contrasting with our findings. Both studies, however, found fair agreement for late decelerations.^5^ Comparisons across other deceleration types is difficult due to differences in classification categories. Interestingly, Devane et al. reported a higher kappa value (*κ* = 0.79) for decelerations among midwives, although they used the 1987 FIGO guidelines, which may explain the discrepancy.^14^

Interestingly, junior midwives demonstrated higher interobserver agreement than senior midwives in nearly all categories. Although these findings differ from previous reports,^5^, ^15^, ^20^ they remain plausible.^5^ Junior midwives, having less clinical experience, may adhere more strictly to guidelines, resulting in greater consistency in classification. In contrast, senior midwives may rely more on experience‐based pattern recognition and clinical judgment, which can introduce greater variability and reduce agreement. However, it is important to emphasize that higher interobserver agreement does not necessarily imply higher accuracy in the identification of fetal acidemia, which was not evaluated in our study.

According to the latest Cochrane review, intrapartum CTG monitoring has been associated with an increase in operative deliveries without a corresponding reduction in cerebral palsy or perinatal mortality.^1^ This is largely due to the lack of objectivity and low interobserver agreement in CTG interpretation, making it a major challenge in labor management. Clinical decisions during labor should be based on a robust and reproducible system; however, our findings suggest that the 2015 FIGO guidelines do not offer sufficient reliability in this regard.

Future research should focus on implementing strategies to address this issue. Although our results do not support the hypothesis that greater experience leads to higher agreement, regular educational programs and structured training for all professionals involved in labor management should be offered and focused on standardized interpretation protocols.^14^, ^21^, ^22^ However, it is becoming increasingly evident that traditional training approaches may not be sufficient. Recent evidence suggests that assessing midwives' CTG interpretation skills through structured evaluations can help identify specific knowledge gaps and guide more effective, tailored training interventions.^23^ Additionally, the development of computerized decision‐support systems or artificial intelligence models may further improve objectivity and reproducibility in CTG classification.^24^, ^25^, ^26^

Some limitations should be acknowledged. First, CTG interpretation was performed outside the clinical setting, meaning that observers were not exposed to real‐time clinical pressures. Second, midwives were provided only with the final 60 min of the CTG tracing prior to the operative delivery. Although we focused on the critical decision making window, we excluded the full progression of labor, which may have influenced CTG interpretation.

Third, detailed deceleration assessments were not obtained from two senior midwives, reducing the number of observers from six to four for this parameter. This reduction affects both the main analysis and subgroup comparisons, potentially introducing bias and limiting generalizability. The reduced and unbalanced sample of observers may have influenced the observed agreement levels, particularly for deceleration subcategories. Finally, the use of non‐consecutive CTG tracings may introduce a degree of selection bias, although we deliberately selected a diverse range of clinical scenarios and CTG patterns to enhance heterogeneity and applicability.

In conclusion, interobserver agreement for intrapartum CTG interpretation using the 2015 FIGO guidelines among midwives was fair. Agreement for individual parameters varied considerably, ranging from poor (variability) to good (decelerations). Interestingly, junior midwives demonstrated a higher level of agreement than their senior counterparts. Future studies should explore strategies to improve interobserver agreement, including education, standardized training programs, and decision‐support technologies.

## AUTHOR CONTRIBUTIONS

Serena Neri: Conceptualization, design, manuscript and submission. Ruben Ramirez Zegarra: Manuscript and supervision. Elisa Nicolini: Literature review, data collection and figures. Francesca Frati: Literature review and data collection. Sara Tagliaferri: Statistical analysis, figures and review. Tullio Ghi: Conceptualization, design, manuscript and supervision.

## FUNDING INFORMATION

The authors did not receive any financial support for this work. No funding has been received for the conduct of this study.

## CONFLICT OF INTEREST STATEMENT

The authors have no conflicts of interest.

## Supporting information


**Table S1.** Baseline and perinatal characteristics of the included women, from whom 100 Cardiotocography traces were selected.

## Data Availability

Research data are not shared.
