# Supplementary material for: Interobserver agreement among midwives in cardiotocography interpretation using the 2015 FIGO guidelines for intrapartum fetal monitoring: A retrospective study
Source: Int J Gynaecol Obstet. 2025 Nov 24;173(2):883–8. doi: 10.1002/ijgo.70687 (PMC13094674; doi:10.1002/ijgo.70687)
Supplement: Supplementary file 1 — Table S1. Baseline and perinatal characteristics of the included women, from whom 100 Cardiotocography traces were selected. [file IJGO-173-883-s001.docx]

Supplementary Table 1. Baseline and perinatal characteristics of the included women, from whom 100 Cardiotocography traces were selected.

| **Variable** | **All cases**  **N=100** |
| --- | --- |
| ***Maternal age, year***  ***mean ± SD*** | 32.4 ± 5.4 |
| ***Parity***  *- Nullipara*  *- Multipara* | 75 (75.0%)  25 (25.0%) |
| ***Mode of delivery***  *- Operative vaginal delivery*  *- Cesarean section* | 60 (60.0%)  40 (40.0%) |
| ***Indication for the operative delivery***  *- Suspected intrapartum fetal compromise*  *- Dystocia of the first stage of labor*  *- Dystocia of the second stage of labor*  *- Non-hypoxic causes* | 46 (46.0%)  22 (22.0%)  29 (29.0%)  3 (3.0%) |
| ***Gestational age at delivery, weeks^+days^***  ***mean ± SD*** | 39^+2^ ± 1^+1^ |
| ***Birthweight, grams***  ***mean ± SD*** | 3439 ± 473 |
| ***Apgar at 5 minutes < 7*** | 5 (5.0%) |
| ***Umbilical artery pH < 7.10*** | 7 (7.0%) |
| ***NICU admission*** | 7 (7.0%) |

*Footnotes. Data are given as mean ± standard deviation (SD), or number (percentage).*

*NICU, neonatal intensive care unit.*
